# On the Treatment of Bilious Colic by Copious Enemata

**Published:** 1846-03

**Authors:** J. S. Paige

**Affiliations:** Oswego, N. Y.


*On the Treatment of Bilious Colic by copious Enemata.* By J. S. Paige, M. D, of Oswego, N. Y.—The case which led to my first essay in this mode of treatment occurred in the summer of 1818, when I was called to attend a man, aged about 35 years, who was laboring under a very severe attack of bilious colic. I pursued the rotine of treatment usually followed in such cases, and called to my aid several reputable practitioners of medicine as counsel, and after using various cathartic and emetic medicines, blood letting, opium, a blister on the abdomen, fomentations, general warm bathing, and oft repeated enemas for forty eight hours or more, I found that I had made no progress towards affording my patient even any hope of relief from his almost intolerable condition, when a lucky thought, as it proved to be, came into my mind —that of making a direct application of some deg ee of pressure upon the constricted portion of the intestine from below, by introducing a large quantity of fluid with a syringe.

I accordingly directed six pints of water, milk, and molasses, which I commenced using with a half pint syringe—the largest at my command at the time—and after using almost the whole of the liquid on hand, the patient began to complain of increased pain and pressure at the point of obstruction, and desired me to desist; but when I made known to him my plan of operations, and that this afforded him the only hope of relief, as I believed, he patiently submitted to my wishes to retain the fluid and allow me to make a little firm pressure therewith on the constriction for a few minutes, which I did, and on withdrawing the syringe, I, as well as the patient, had the great satisfaction to find that the object of our most earnest desires was accomplished; for copious discharges of fæcal matter took place in a very short time, and an almost immediate relief was the result, only a considerable degree of tenderness and some fever remaining, which, however, soon subsided by the use of mild laxatives, diaphoretics, &c.

Two or three weeks afterwards the same individual had another attack of the same disease, from his own imprudence. I commenced the treatment by overcoming the obstruction in the same way as at first, which succeeded in a very few minutes in giving relief; three quarts of fluid also were used on this occasion.

The success of this plan in these two cases, induced me to commence the treatment of similar cases in the same way, and it has been the uniform mode of my treatment ever since (except in one case of a female where delicacy required a short trial of the ordinary means, and which were soon successful in that case,) and I have never failed in affording speedy relief in any case.

In two cases occurring in another individual, I found it necessary to use six quarts each time before relief was obtained; but I have never found it necessary to introduce more than about three quarts in any case, except in the ones last mentioned.

Since the compound syringe has been introduced into use, we are enabled to force liquids into the intestines with more facility than with the common syringe, and therefore whenever it becomes necessary to use large quantities in this way for any purpose, we have more efficient means of doing it at our command.

The quantity of fluid to be used in these cases must be measured by the demand of each individual case, and the directions I would give for securing the object in view, are, to introduce, on our first arrival at a case of this kind, a quantity of mild liquid, such as warm water, or warm water mixed with molasses, or some mucilaginous fluid, slowly and steadily till the obstruction be reached, and then to keep the instrument, in stitu, a few minutes, and make a gentle but firm pressure with the liquid upon the constriction, for if this precaution be not observed, we may fail of affording the relief we so much desire.

After the bowels are evacuated, and the obstruction overcome, some mild laxative should be given to gently move the bowels, and this will generally finish the work; and for this purpose a dose of olive or castor oil, I suppose, will be as good as anything, unless the disease depended on a derangement of the liver, as is often the case, and then more active medicines should be used, at the discretion of the physician.

Some of the considerations which urge me to recommend this mode of treatment are, that relief may be more promptly and surely obtained than by any other method, for although other means may act in time, there must necessarily be some delay, if they be not rejected by vomiting, which usually attends the disease.

Great certainty and promptness, these are strong recommendations in favor of the mechanical treatment, and from what I have seen, I feel very confident that it will succeed in almost all *curable cases*, if seasonably applied, and there is perfect safety also to recommend it, if used with reasonable caution. The use of large injections may serve other valuable purposes—they may be resorted to in removing intestinal concretions, hardened fæces in obstinate constipation, in reducing intussusception of the bowels, perhaps in removing worms in some cases, also in irritated conditions of the mucous membrane, in dysentery or other inflammations, and in some cases of poisons in the bowels.

From the great extent of the digestive and absorbent tube and its many diseases, I think it not improbable that much may be done by large injections as media of conveying medicinal agents where they may be directly applied in the treatment of diseases in that canal.—*N. Y. Jour of Med.*

